# The origin, transmission and clinical therapies on coronavirus disease 2019 (COVID-19) outbreak – an update on the status

**DOI:** 10.1186/s40779-020-00240-0

**Published:** 2020-03-13

**Authors:** Yan-Rong Guo, Qing-Dong Cao, Zhong-Si Hong, Yuan-Yang Tan, Shou-Deng Chen, Hong-Jun Jin, Kai-Sen Tan, De-Yun Wang, Yan Yan

**Affiliations:** 1 Guangdong Provincial Key Laboratory of Biomedical Imaging and Guangdong Provincial Engineering Research Center of Molecular Imaging, Zhuhai, 519000 Guangdong China; 2 Department of Cardiothoracic Surgery, the Fifth Affiliated Hospital, Sun Yat-Sen University, Zhuhai, 519000 Guangdong China; 3 Center of Infectious Disease, the Fifth Affiliated Hospital, Sun Yat-Sen University, Zhuhai, 519000 Guangdong China; 4 Department of Otolaryngology, Yong Loo Lin School of Medicine, National University of Singapore, National University Health System, Singapore, 119228 Singapore; 5 Center for Interventional Medicine, the Fifth Affiliated Hospital, Sun Yat-Sen University, Zhuhai, 519000 Guangdong China

**Keywords:** Clinical characteristics, Coronavirus disease 2019 (COVID-19), Origin, SARS-CoV-2, Therapy, Transmission

## Abstract

An acute respiratory disease, caused by a novel coronavirus (SARS-CoV-2, previously known as 2019-nCoV), the coronavirus disease 2019 (COVID-19) has spread throughout China and received worldwide attention. On 30 January 2020, World Health Organization (WHO) officially declared the COVID-19 epidemic as a public health emergency of international concern. The emergence of SARS-CoV-2, since the severe acute respiratory syndrome coronavirus (SARS-CoV) in 2002 and Middle East respiratory syndrome coronavirus (MERS-CoV) in 2012, marked the third introduction of a highly pathogenic and large-scale epidemic coronavirus into the human population in the twenty-first century. As of 1 March 2020, a total of 87,137 confirmed cases globally, 79,968 confirmed in China and 7169 outside of China, with 2977 deaths (3.4%) had been reported by WHO. Meanwhile, several independent research groups have identified that SARS-CoV-2 belongs to β-coronavirus, with highly identical genome to bat coronavirus, pointing to bat as the natural host. The novel coronavirus uses the same receptor, angiotensin-converting enzyme 2 (ACE2) as that for SARS-CoV, and mainly spreads through the respiratory tract. Importantly, increasingly evidence showed sustained human-to-human transmission, along with many exported cases across the globe. The clinical symptoms of COVID-19 patients include fever, cough, fatigue and a small population of patients appeared gastrointestinal infection symptoms. The elderly and people with underlying diseases are susceptible to infection and prone to serious outcomes, which may be associated with acute respiratory distress syndrome (ARDS) and cytokine storm. Currently, there are few specific antiviral strategies, but several potent candidates of antivirals and repurposed drugs are under urgent investigation. In this review, we summarized the latest research progress of the epidemiology, pathogenesis, and clinical characteristics of COVID-19, and discussed the current treatment and scientific advancements to combat the epidemic novel coronavirus.

## Background

In December 2019, a cluster of pneumonia cases, caused by a newly identified β-coronavirus, occurred in Wuhan, China. This coronavirus, was initially named as the 2019-novel coronavirus (2019-nCoV) on 12 January 2020 by World Health Organization (WHO). WHO officially named the disease as coronavirus disease 2019 (COVID-19) and Coronavirus Study Group (CSG) of the International Committee proposed to name the new coronavirus as SARS-CoV-2, both issued on 11 February 2020. The Chinese scientists rapidly isolated a SARS-CoV-2 from a patient within a short time on 7 January 2020 and came out to genome sequencing of the SARS-CoV-2 [1]. As of 1 March 2020, a total of 79,968 cases of COVID-19 have been confirmed in mainland China including 2873 deaths [2]. Studies estimated the basic reproduction number (*R*_*0*_) of SARS-CoV-2 to be around 2.2 [3], or even more (range from 1.4 to 6.5) [4], and familial clusters of pneumonia [5] outbreaks add to evidence of the epidemic COVID-19 steadily growing by human-to-human transmission.

## Origin and transmission of SARS-CoV-2

**Fig. 1:**
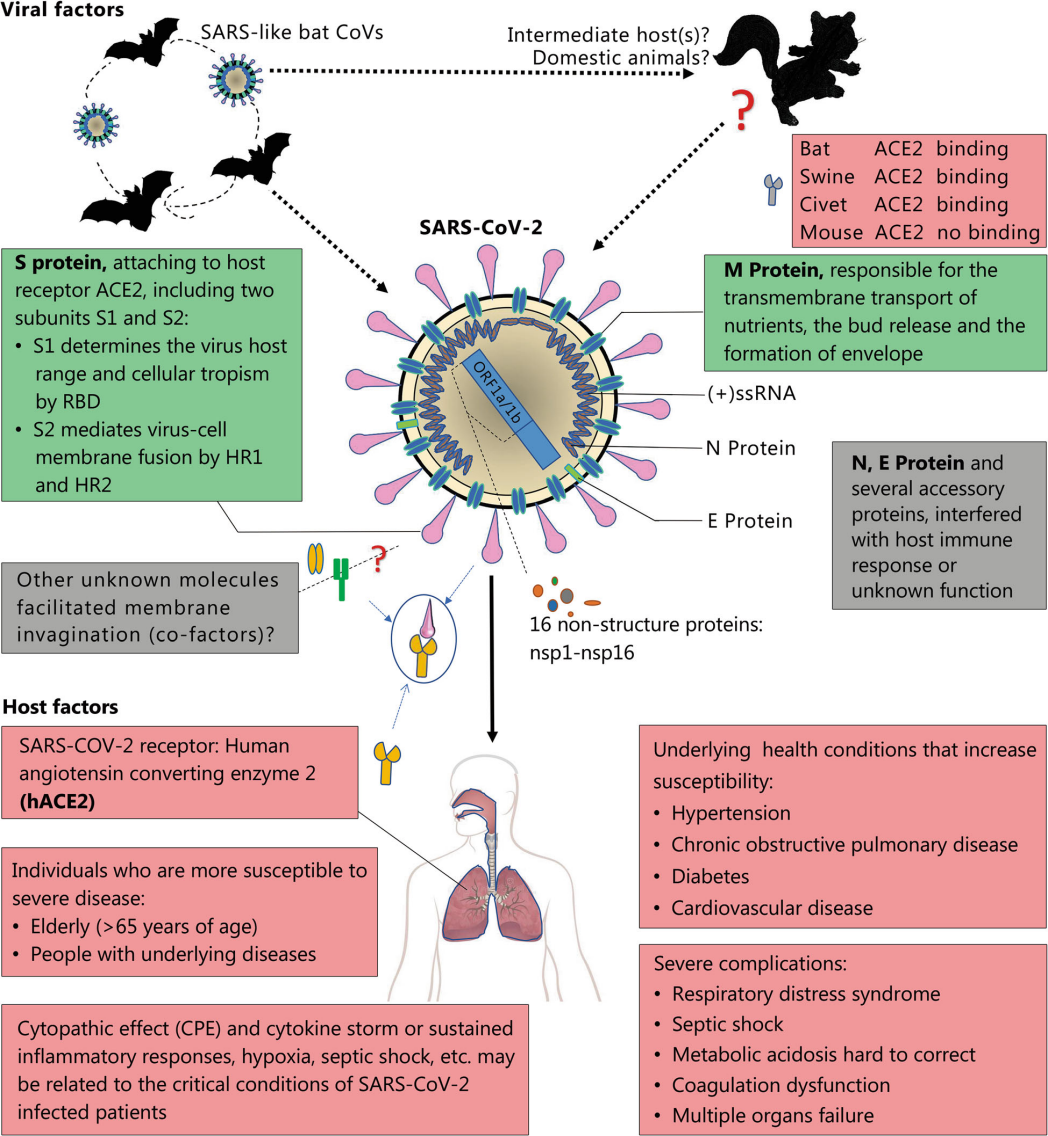
Viral and host factors that influence the pathogenesis of SARS-CoV-2. Bats are the reservoir of a wide variety of coronaviruses, including severe acute respiratory syndrome coronavirus (SARS-CoV) -like viruses. SARS-CoV-2 may originate from bats or unknown intermediate hosts and cross the species barrier into humans. Virus-host interactions affect viral entry and replication. Upper panel: Viral factor. SARS-CoV-2 is an enveloped positive single-stranded RNA (ssRNA) coronavirus. Two-thirds of viral RNA, mainly located in the first open reading frame (ORF 1a/b), encodes 16 non-structure proteins (NSPs). The rest part of the virus genome encodes four essential structural proteins, including spike (S) glycoprotein, small envelope (E) protein, matrix (M) protein, and nucleocapsid (N) protein, and also several accessory proteins. S glycoprotein of SARS-CoV-2 binds to host cell receptors, angiotensin-converting enzyme 2 (ACE2), that is a critical step for virus entry. The possible molecules facilitated membrane invagination for SARS-CoV-2 endocytosis are still unclear. Other virus proteins may contribute to pathogenesis. Host factors (Lower panel) can also influence susceptibility to infection and disease progression. The elderly and people with underlying disease are susceptible to SARS-CoV-2 and tend to develop into critical conditions. RBD, receptor-binding domain; HR1, heptad repeats 1; HR2, heptad repeats 2

The SARS-CoV-2 is a β-coronavirus, which is enveloped non-segmented positive-sense RNA virus (subgenus *sarbecovirus*, *Orthocoronavirinae* subfamily) [6]. Coronaviruses (CoV) are divided into four genera, including α−/β−/γ−/δ-CoV. α- and β-CoV are able to infect mammals, while γ- and δ-CoV tend to infect birds. Previously, six CoVs have been identified as human-susceptible virus, among which α-CoVs HCoV-229E and HCoV-NL63, and β-CoVs HCoV-HKU1 and HCoV-OC43 with low pathogenicity, cause mild respiratory symptoms similar to a common cold, respectively. The other two known β-CoVs, SARS-CoV and MERS-CoV lead to severe and potentially fatal respiratory tract infections [7]. It was found that the genome sequence of SARS-CoV-2 is 96.2% identical to a bat CoV RaTG13, whereas it shares 79.5% identity to SARS-CoV. Based on virus genome sequencing results and evolutionary analysis, bat has been suspected as natural host of virus origin, and SARS-CoV-2 might be transmitted from bats via unknown intermediate hosts to infect humans. It is clear now that SARS-CoV-2 could use angiotensin-converting enzyme 2 (ACE2), the same receptor as SARS-CoV [8], to infect humans (upper panel, Fig. 1).

### Epidemiology − reservoirs and transmission

The epidemic of unknown acute respiratory tract infection broke out first in Wuhan, China, since 12 December 2019, possibly related to a seafood market. Several studies suggested that bat may be the potential reservoir of SARS-CoV-2 [9, 10]. However, there is no evidence so far that the origin of SARS-CoV-2 was from the seafood market. Rather, bats are the natural reservoir of a wide variety of CoVs, including SARS-CoV-like and MERS-CoV-like viruses [11–13]. Upon virus genome sequencing, the COVID-19 was analyzed throughout the genome to Bat CoV RaTG13 and showed 96.2% overall genome sequence identity [8], suggesting that bat CoV and human SARS-CoV-2 might share the same ancestor, although bats are not available for sale in this seafood market [14]. Besides, protein sequences alignment and phylogenetic analysis [15] showed that similar residues of receptor were observed in many species, which provided more possibility of alternative intermediate hosts, such as turtles, pangolin and snacks.

Human-to-human transmission of SARS-CoV-2 occurs mainly between family members, including relatives and friends who intimately contacted with patients or incubation carriers. It is reported [16] that 31.3% of patients recent travelled to Wuhan and 72.3% of patients contacting with people from Wuhan among the patients of non-residents of Wuhan. Transmission between healthcare workers occurred in 3.8% of COVID-19 patients, issued by the National Health Commission of China on 14 February 2020. By contrast, the transmission of SARS-CoV and MERS-CoV is reported to occur mainly through nosocomial transmission. Infections of healthcare workers in 33–42% of SARS cases and transmission between patients (62–79%) was the most common route of infection in MERS-CoV cases [17, 18]. Direct contact with intermediate host animals or consumption of wild animals was suspected to be the main route of SARS-CoV-2 transmission. However, the source(s) and transmission routine(s) of SARS-CoV-2 remain elusive.

### Genome structure and key viral factors

Isolated from a COVID-19 pneumonia patient, a worker in the Wuhan seafood market, the complete genome of Wuhan-Hu-1 coronavirus (WHCV), one strain of SARS-CoV-2, is 29.9 kb [14]. While SARS-CoV and MERS-CoV have positive-sense RNA genomes of 27.9 kb and 30.1 kb, respectively [19]. It has been shown that the genome of CoVs contains a variable number (6–11) of open reading frames (ORFs) [20]. Two-thirds of viral RNA, mainly located in the first ORF (ORF1a/b) translates two polyproteins, pp1a and pp1ab, and encodes 16 non-structural proteins (NSP), while the remaining ORFs encode accessory and structural proteins. The rest part of virus genome encodes four essential structural proteins, including spike (S) glycoprotein, small envelope (E) protein, matrix (M) protein, and nucleocapsid (N) protein [21], and also several accessory proteins, that interfere with the host innate immune response. Wu et al. [14] have recently performed deep meta-transcriptomic sequencing on WHCV, which contained 16 predicted NSP. WHCV exhibits some genomic and phylogenetic similarity to SARS-CoV, particularly in the S-glycoprotein gene and receptor-binding domain (RBD), indicating the capability of direct human transmission. Compared with the known SARS-CoV and MERS-CoV genome, SARS-CoV-2 is closer to the SARS-like bat CoVs in terms of the whole genome sequence. Most genomic encoded proteins of SARS-CoV-2 are similar to SARS-CoVs, as well as exist certain differences. At the protein level, there are no amino acid substitutions that occurred in NSP7, NSP13, envelope, matrix, or accessory proteins p6 and 8b, except in NSP2, NSP3, spike protein, underpinning subdomain, i.e., RBD [22]. Another recent research suggested [23] that the mutation in NSP2 and NSP3 play a role in infectious capability and differentiation mechanism of SARS-CoV-2. This provokes people to explore the difference of the host tropism and transmission between SARS-CoV-2 and SARS-CoV or conduct further investigations on the potential therapeutic targets. Zhang et al. [24] analyzed the genotypes of COVID-19 in different patients from several provinces and found that SARS-CoV-2 had been mutated in different patients in China. Although the degree of diversification of SARS-CoV-2 is smaller than the mutation of H7N9 avian influenza [25]. Tang et al. [26] conducted a population genetic analyses of 103 SARS-CoV-2 genomes and classified out two prevalent evolvement types of SARS-CoV-2, L type (~ 70%) and S type (~ 30%). The strains in L type, derived from S type, are evolutionarily more aggressive and contagious. Thus, virologists and epidemiologists need to closely monitor the novel coronavirus, in order to inspect the virulence and epidemic.

### Coronavirus replication and pathogenesis

ACE2, found in the lower respiratory tract of humans, is known as cell receptor for SARS-CoV [27] and regulates both the cross-species and human-to-human transmission [28]. Isolated from the bronchoalveolar lavage fluid (BALF) of a COVID-19 patient, Zhou et al. [8] have confirmed that the SARS-CoV-2 uses the same cellular entry receptor, ACE2, as SARS-CoV. The virion S-glycoprotein on the surface of coronavirus can attach to the receptor, ACE2 on the surface of human cells [29]. S glycoprotein includes two subunits, S1 and S2 [30]. S1 determines the virus-host range and cellular tropism with the key function domain − RBD, while S2 mediates virus-cell membrane fusion by two tandem domains, heptad repeats 1 (HR1) [31] and HR2 [32]. After membrane fusion, the viral genome RNA is released into the cytoplasm, and the uncoated RNA translates two polyproteins, pp1a and pp1ab [33], which encode non-structural proteins, and form replication-transcription complex (RTC) in double-membrane vesicle [34]. Continuously RTC replicate and synthesize a nested set of subgenomic RNAs [35], which encode accessory proteins and structural proteins. Mediating endoplasmic reticulum (ER) and Golgi [36], newly formed genomic RNA, nucleocapsid proteins and envelope glycoproteins assemble and form viral particle buds. Lastly, the virion-containing vesicles fuse with the plasma membrane to release the virus.

Because the binding of SARS-CoV-2 Spike (S) glycoprotein and ACE2 receptor is a critical step for virus entry, virus-receptor binding affinity is under intensive study through different approaches. Systematic detection of β-CoV receptors showed that human cells expressing ACE2, but not human Dipeptidyl peptidase-4 (DPP4) or APN (Aminopeptidase N), were enhanced entry of SARS-CoV-2 [37]. While, another study showed that S-protein and ACE2 binding efficiency is 10- to 20- fold higher than that of SARS-CoV, evidenced by Cryo-EM Structure of the SARS-CoV-2 Spike in the prefusion conformation [38]. For SARS-CoV, the cleavage of trimer S protein is triggered by the cell surface-associated transmembrane protease serine 2 (TMPRSS2) [39] and cathepsin [40], while the possible molecules facilitated membrane invagination for SARS-CoV-2 endocytosis are still unclear. Up to the date this review paper was prepared, reports showed that the SARS-CoV-2 may readily transmit, while cause less serious human infection rather than human SARS-CoV. Based on the latest WHO report, the number of infected people (over 80,000 globally, updated on 1 March 2020). The global outbreak may due to the following factors: firstly, the unknown pneumonia outbroke at the time of China Spring Festival, when the mass population flowing. Secondly, more detailed molecular mechanisms of viral binding and entry manners await to be elucidated, which may hamper the development of targeted therapy. Thirdly, available data suggested that the SARS-CoV-2 may be less virulent than the SARS-CoV and MERS-CoV, with the currently analyzed mortality of COVID-19 is 3.4%, lower than death rate of SARS (9.6%) and MERS (around 35%), respectively [19]. Thus, the potential mechanisms for human-to-human transmission and pathogenic mechanisms of the SARS-CoV-2 are under extensively studied.

## Clinical characteristics

As an emerging acute respiratory infectious disease, COVID-19 primarily spreads through the respiratory tract, by droplets, respiratory secretions, and direct contact [41] for a low infective dose [42]. Otherwise, it has been reported a SARS-CoV-2 was isolated from fecal swabs of a severe pneumonia patient on 10 February 2020 from a critical case in the Fifth Affiliated Hospital, Sun Yat-Sen University, Guangdong, China. Likewise, Zhang et al. [43] have found the presence of SARS-CoV-2 in fecal swabs and blood, indicating the possibility of multiple routes transmission. ACE2 protein presents in abundance on lung alveolar epithelial cells and enterocytes of small intestine remarkably [44], which may help understand the routes of infection and disease manifestations. Based on current epidemiological investigation, the incubation period is 1–14 days, mostly 3–7 days. And the COVID-19 is contagious during the latency period [45]. It is highly transmissible in humans, especially in the elderly and people with underlying diseases. The median age of patients is 47–59 years, and 41.9–45.7% of patients were females [16, 41, 46]. As it is designated SARS-CoV-2, COVID-19 patients presented certainly similar symptoms, such as fever, malaise, and cough [47]. Most adults or children with SARS-CoV-2 infection presented with mild flu-like symptoms and a few patients are in critical condition and rapidly develop acute respiratory distress syndrome, respiratory failure, multiple organ failure, even deaths [48].

### Diagnostic criteria

The viral research institution in China has conducted preliminary identification of the SARS-CoV-2 through the classical Koch’s postulates and observing its morphology through electron microscopy [49]. So far, the golden clinical diagnosis method of COVID-19 is nucleic acid detection in the nasal and throat swab sampling or other respiratory tract samplings by real-time PCR and further confirmed by next-generation sequencing.

### Clinical symptoms

A recent study led by Prof. Nan-Shan Zhong’s team, by sampling 1099 laboratory-confirmed cases, found that the common clinical manifestations included fever (88.7%), cough (67.8%), fatigue (38.1%), sputum production (33.4%), shortness of breath (18.6%), sore throat (13.9%), and headache (13.6%) [16]. In addition, a part of patients manifested gastrointestinal symptoms, with diarrhea (3.8%) and vomiting (5.0%). The clinical manifestations were in consistence with the previous data of 41, 99, and 138 patients analysis in Hubei province [46, 48, 50]. Fever and cough were the dominant symptoms whereas upper respiratory symptoms and gastrointestinal symptoms were rare, suggesting the differences in viral tropism as compared with SARS-CoV [51], MERS-CoV [52], and influenza [53]. The elderly and those with underlying disorders (i.e., hypertension, chronic obstructive pulmonary disease, diabetes, cardiovascular disease), developed rapidly into acute respiratory distress syndrome, septic shock, metabolic acidosis hard to correct and coagulation dysfunction, even leading to the death [48] (lower panel, Fig. 1).

In laboratory examination results, most patients had normal or decreased white blood cell counts, and lymphocytopenia [16, 54]. But in the severe patients, the neutrophil count, D-dimer, blood urea, and creatinine levels were higher significantly, and the lymphocyte counts continued to decrease. Additionally, inflammatory factors (interleukin (IL)-6, IL-10, tumor necrosis factor-α (TNF-α) increase, indicating the immune status of patients. The data showed that ICU patients had higher plasma levels of IL-2, IL-7, IL-10, granulocyte colony-stimulating factor (GCSF), 10 kD interferon-gamma-induced protein (IP-10), monocyte chemoattractant protein-1 (MCP-1), macrophage inflammatory protein 1-α (MIP-1α), and TNF-α [48].

Moreover, the CT imaging showed that computed tomography on the chest was ground-glass opacity (56.4%) and bilateral patchy shadowing (51.8%) [16], sometimes with a rounded morphology and a peripheral lung distribution, analyzed from the patients in the Fifth Affiliated Hospital, Sun Yat-Sen University [55]. Clinicians have been aware that, a part of confirmed patients appeared the normal CT image presentations. The diagnostic sensitivity of radiologic is limited, so it is necessary to verify with clinical symptoms and virus RNA detections.

### Complications and clinical outcomes

Based on the current information, most patients had a good prognosis, while a few patients were in critical condition, especially the elderly and those with chronic underlying diseases. As of 1 March 2020, a total of 79,968 confirmed cases, including 14,475 (18.1%) with severe illness, and 2873 deaths (3.5%) in mainland China had been reported by WHO [2]. Complications included acute respiratory distress syndrome (ARDS), arrhythmia, shock [46], acute kidney injury, acute cardiac injury, liver dysfunction and secondary infection [48]. The poor clinical outcome was related to disease severity. The disease tends to progress faster in elderly people, with the median number of days from the occurrence of the first symptoms to death shorter among people aged 65 years or more [56, 57]. Similar to H7N9 patients [58], the elderly male with comorbidities and ARDS showed a higher death risk. Additionally, more than 100 children were infected, with the youngest being 30 h after birth [59]. Neonates and the elderly need more attention and care due to their immature or weak immune system.

## Host immune response and immunopathology

The immune response is vital for the control and resolution of CoV infections, while it can also lead to immunopathogenesis, associated with the immune response out of control. The S proteins of Coronavirus binds to the host cells by ACE2, fusing to the membrane and release the viral RNA. The viral RNAs, as pathogen-associated molecular patterns (PAMPs), are detected by the pattern recognition receptors (PRRs). Usually, Toll-like receptor (TLR) 3, TLR7, TLR8, and TLR9 sense viral RNA and DNA in the endosome [60, 61]. The viral RNA receptor retinoic-acid inducible gene I (RIG-I) [62], cytosolic receptor melanoma differentiation-associated gene 5 (MDA5) and nucleotidyltransferase cyclic GMP-AMP synthase (cGAS) [63] are responsible for the recognition of viral RNA and DNA in the cytoplasm. These complex signalling recruit adaptors, including TIR-domain-containing adaptor protein including IFN-β (TRIF), mitochondrial antiviral-signalling protein (MAVS) [64] and stimulator of interferon genes protein (STING) [65] to trigger downstream cascades molecules, involving adaptor molecule MyD88 and lead to the activation of the transcription factor nuclear factor-κB (NF-κB) and interferon regulatory factor 3 (IRF3) and the production of type I Interferons (IFN-α /β) and a series of pro-inflammatory cytokines [66]. Hence, virus-cell interactions produce a diverse set of immune mediators against the invading virus [67]. Innate immunity is needed in a precise regulation to eliminate the virus, otherwise will result in immunopathology. A few plasma cytokines and chemokines were observed ascended in COVID-19 patients, including IL-1, IL-2, IL-4, IL-7, IL-10, IL-12, IL-13, IL-17, GCSF, macrophage colony-stimulating factor (MCSF), IP-10, MCP-1, MIP-1α, hepatocyte growth factor (HGF), IFN-γ and TNF-α [48, 68, 69]. Of note, an anatomy report of COVID-19 pneumonia corpse [70] indicated that COVID-19 caused an inflammatory response in the lower airway and led to lung injury. Collectively, the virus particles invade the respiratory mucosa firstly and infect other cells, triggering a series of immune responses and the production of cytokine storm in the body, which may be associated with the critical condition of COVID-19 patients.

## Treatment of COVID-19

### Current therapies

Given the lack of effective antiviral therapy against COVID-19, current treatments mainly focused on symptomatic and respiratory support according to the Diagnosis and Treatment of Pneumonia Caused by COVID-19 (updated to version 6) issued by National Health Commission of the People’s Republic of China [71]. Nearly all patients accepted oxygen therapy, and WHO recommended extracorporeal membrane oxygenation (ECMO) to patients with refractory hypoxemia [72]. Rescue treatment with convalescent plasma and immunoglobulin G [73] are delivered to some critical cases according to their conditions.

### Antiviral treatments

Based on the experience of fighting the epidemic SARS-CoV and MERS-CoV previously, we may learn some lessons for some treatment strategies against coronavirus [74]. Antiviral drugs and systemic corticosteroid treatment commonly used in clinical practice previously, including neuraminidase inhibitors (oseltamivir, peramivir, zanamivir, etc), ganciclovir, acyclovir, and ribavirin, as well as methylprednisolone [46, 75] for influenza virus, are invalid for COVID-19 and not recommended. Remdesivir (GS-5734) is a 1′-cyano-substituted adenosine nucleotide analog prodrug and shows broad-spectrum antiviral activity against several RNA viruses. Based on the data collected from in vitro cell line and mouse model, remdesivir could interfere with the NSP12 polymerase even in the setting of intact ExoN proofreading activity [76]. Remdesivir has been reported to treat the first US case of COVID-19 successfully [77]. Chloroquine is a repurposed drug with great potential to treat COVID-19. Chloroquine has been used to treat malaria for many years [78], with a mechanism that is not well understood against some viral infections. Several possible mechanisms are investigated: Chloroquine can inhibit pH-dependent steps of the replication of several viruses [79], with a potent effect on SARS-CoV infection and spread [80]. Moreover, chloroquine has immunomodulatory effects, suppressing the production/release of TNF-α and IL-6. It also works as a novel class of autophagy inhibitor [81], which may interfere with viral infection and replication. Several studies have found that chloroquine interfered with the glycosylation of cellular receptors of SARS-CoV [80] and functioned at both entry and at post-entry stages of the COVID-19 infection in Vero E6 cells [82]. A combination of remdesivir and chloroquine was proven to effectively inhibit the recently emerged SARS-CoV-2 in vitro.

Scientists previously confirmed that the protease inhibitors lopinavir and ritonavir, used to treat infection with human immunodeficiency virus (HIV) [83], could improve the outcome of MERS-CoV [84] and SARS-CoV [85] patients. It has reported that β-coronavirus viral loads of a COVID-19 patient in Korea significantly decreased after lopinavir/ritonavir (Kaletra®, AbbVie, North Chicago, IL, USA) treatment [86]. Additionally, clinicians combined Chinese and Western medicine treatment including lopinavir/ritonavir (Kaletra®), arbidol, and Shufeng Jiedu Capsule (SFJDC, a traditional Chinese medicine) and gained significant improvement in pneumonia associated symptoms in Shanghai Public Health Clinical Center, China [87].The other antiviral drugs include nitazoxanide, favipiravir, nafamostat, and so on (See Table 1 for details).

**Table 1:** Common and potent antiviral drugs

Status	Drugs	Action mode	Anti-infective mechanism	Target diseases	Ref.
Approved	Lopinavir/Ritonavir	Protease inhibitors	Inhibiting HIV-1 protease for protein cleavage, resulting in non-infectious, immature viral particles	HIV/AIDS, SARS, MERS	[83–85]
Approved, Investigational, Vet approved	Chloroquine	9-aminoquinolin	Increasing endosomal pH, immunomodulating, autophagy inhibitors	Malaria, autoimmune disease	[79–82]
Experimental	Remdesivir (GS-5734)	Nucleotide analogue prodrug	Interfering with virus post-entry	Ebola, SARS, MERS (A wide array of RNA viruses)	[76, 88, 89]
Investigational	Nafamostat	Synthetic serine protease inhibitor	Prevents membrane fusion by reducing the release of cathepsin B; anticoagulant activities	Influenza, MERS, Ebola	[90, 91]
Approved	Ribavirin	Synthetic guanosine nucleoside	Interfering with the synthesis of viral mRNA (a broad-spectrum activity against several RNA and DNA viruses)	HCV, SARS, MERS	[92–94]
Approved	Oseltamivir	Neuraminidase inhibitor	Inhibiting the activity of the viral neuraminidase enzyme, preventing budding from the host cell, viral replication, and infectivity	Influenza viruses A	[95, 96]
Approved	Penciclovir/ Acyclovir	Nucleoside analog	A synthetic acyclic guanine derivative, resulting in chain termination	HSV, VZV	[97]
Approved, Investigational	Ganciclovir	Nucleoside analog	Potent inhibitor of the Herpesvirus family including cytomegalovirus	AIDS-associated cytomegalovirus infections	[98]
Investigational	Favipiravir (T-705)	Nucleoside analog: Viral RNA polymerase inhibitor	Acting on viral genetic copying to prevent its reproduction, without affecting host cellular RNA or DNA synthesis	Ebola, influenza A(H1N1)	[99–101]
Approved, Investigational, Vet approved	Nitazoxanide	Antiprotozoal agent	Modulating the survival, growth, and proliferation of a range of extracellular and intracellular protozoa, helminths, anaerobic and microaerophilic bacteria, viruses	A wide range of viruses including human/animal coronaviruses	[102–104]

*HIV* Human immunodeficiency virus, *AIDS* Acquired immune deficiency syndrome, *SARS* Severe acute respiratory syndrome, *MERS* Middle East respiratory syndrome, *HCV* Hepatitis C virus, *HSV* Herpes simplex virus, *VZV* Varicella-zoster virus

## Conclusions

The outbreak of COVID-19 swept across China rapidly and has spread to 85 countries/territories/areas outside of China as of 5 March 2020 [2]. Scientists have made progress in the characterization of the novel coronavirus and are working extensively on the therapies and vaccines against the virus. We have summarized the current knowledge of SARS-CoV-2 as follows: Firstly, the emerging pneumonia, COVID-19, caused by SARS-CoV-2, exhibits strong infectivity but less virulence, compared to SARS and MERS, in terms of morbidity and mortality. Originating from reservoir of bats and unknown intermediate hosts, SARS-CoV-2 binds to ACE2 with high affinity as a virus receptor to infect humans. Secondly, the susceptible population involves the elderly and people with certain underlying medical conditions, which requires more attention and care. Thirdly, so far, the supporting treatments, combined with potent antiviral drugs, such as remdesivir, chloroquine, or lopinavir/ritonavir, have been conducted with definite effect on treat COVID-19 patients, while solid data from more clinical trials are needed. However, questions remain vague and more studies are urgent to explore the transmission and pathogenicity mechanism of the emerging coronavirus. To make clear the evolutionary path from the original host to cross-species transmission so as to potentially limit the transmission to naïve animals or humans. In addition, to uncover the mystery of the molecular mechanism of viral entry and replication, which provides the basis of future research on developing targeted antiviral drugs and vaccines.

Given more than 80% of patients are confirmed in Hubei province, the hospitals and medical workers in Hubei are facing and bearing enormous pressure and severe challenge, including a high risk of infection and inadequate protection, as well as overwork, frustration and exhaustion [105]. Chinese Government and authorities have launched psychological intervention, and we sincerely hope that Chinese people and other countries overcome the epidemic as fast as possible.

## Data Availability

The data and materials used during the current review are all available in this review.

## Abbreviations

ACE2: Angiotensin-converting enzyme 2
APN: Aminopeptidase N
ARDS: Acute respiratory distress syndrome
BALF: Bronchoalveolar lavage fluid
cGAS: Cyclic GMP-AMP synthase
CoV: Coronavirus
COVID-19: Coronavirus disease 2019
CSG: Coronavirus Study Group
DPP4: Dipeptidyl peptidase-4
E protein: Envelope protein
ECMO: Extracorporeal membrane oxygenation
ER: Endoplasmic reticulum
GCSF: Granulocyte colony-stimulating factor
HGF: Hepatocyte growth factor
HR1: Heptad repeats 1
HR2: Heptad repeats 2
IFN: Interferon
IL: Interleukin
IP-10: 10 kD interferon-gamma-induced protein
IRF3: Interferon regulatory factor 3
M protein: Matrix protein
MAVS: Mitochondrial antiviral-signalling protein
MCP-1: Monocyte chemoattractant protein-1
MCSF: Macrophage colony-stimulating factor
MDA5: Melanoma differentiation-associated gene 5
MERS-CoV: Middle East respiratory syndrome coronavirus
MIP-1α: Macrophage inflammatory protein 1-α
N protein: Nucleocapsid protein
NF-κB: Nuclear factor-κB
NSP: Non-structure protein
ORF: Open reading frame
PAMP: Pathogen-associated molecular pattern
PRR: Pattern recognition receptor
RBD: Receptor-binding domain
RIG-I: Retinoic-acid inducible gene I
RTC: Replication-transcription complex
S protein: Spike glycoprotein
SARS-CoV: Severe acute respiratory syndrome coronavirus
STING: Stimulator of interferon genes protein
TLR: Toll-like receptor
TMPRSS2: Transmembrane protease serine 2
TNF-α: Tumor necrosis factor α
TRIF: TIR-domain-containing adaptor protein including IFN-β
WHCV: Wuhan-Hu-1 coronavirus
WHO: World Health Organization
